# Self‐aggregation of zinc bacteriochlorophyll‐*d* analogs with an acylhydrazone moiety as the 13‐keto‐carbonyl alternative

**DOI:** 10.1111/php.13949

**Published:** 2024-04-06

**Authors:** Satoru Fujii, Hitoshi Tamiaki

**Affiliations:** ^1^ Graduate School of Life Sciences Ritsumeikan University Kusatsu Shiga Japan

**Keywords:** chlorophyll, chlorosome, electronic absorption spectrum, J‐aggregate, substitution effect

## Abstract

Zinc methyl 3‐hydroxymethyl‐pyropheophorbides‐*a* possessing an acylhydrazinylidene group at the 13^1^‐position were prepared by chemically modifying chlorophyll‐*a*, which were models of bacteriochlorophyll‐*d* as one of the light‐harvesting pigments in photosynthetic green bacteria. Similar to the self‐aggregation of natural bacteriochlorophyll‐*d* in the antenna systems called chlorosomes, some of the synthetic models self‐aggregated in an aqueous Triton X‐100 solution to give red‐shifted and broadened visible absorption bands. The newly appeared oligomeric bands were ascribable to the exciton coupling of the chlorin π‐systems along the molecular *y*‐axis, leading to intense circular dichroism bands in the red‐shifted Qy and Soret regions. The self‐aggregation in the aqueous micelle was dependent on the steric size of the terminal substituent at the 13‐acylhydrazone moiety. An increase in the length of the oligomethylene chain as the terminal moved the red‐shifted Qy maxima to shorter wavelengths, and branched alkyl and benzyl substitutes afforded no more self‐aggregates to leave monomeric species in the hydrophobic environment inside the micelle. These results indicated that the acyl groups on the 13‐hydrazone as the alternative of the natural 13‐ketone regulated the chlorosome‐like self‐aggregation.

## Graphical abstract

Zinc bacteriochlorophyll‐*d* analogs possessing an acylhydrazone moiety as the 13‐keto‐carbonyl alternative were prepared. Similar to self‐aggregation in chlorosomes, synthetic models with R=(CH_2_)_1~6_H self‐aggregated in an aqueous Triton X‐100 solution to give red‐shifted and broadened visible absorption bands with intense CD bands. An increase in the oligomethylene chain length moved the red‐shifted Qy maxima to shorter wavelengths, and branched alkyl and benzyl substitutes have left monomeric species in the hydrophobic environment inside the micelle. The R‐dependent self‐aggregation was ascribable to the specific Zn^…^O3^2^–H^…^N=C13^1^ bond and π–π interaction of the composite chlorin chromophore along the molecular *y*‐axis.

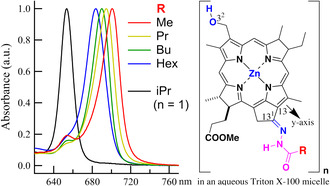

**Abbreviations**
BChl: bacteriochlorophyll
CD: circular dichroism
Chl: chlorophyll
ESI: electrospray ionization
FCC: flash column chromatography
HPLC: high‐performance liquid chromatography
HRMS: high‐resolution mass spectra
NOESY: nuclear Overhauser effect spectroscopy
RP: reverse‐phase
TX‐100: Triton X‐100

## INTRODUCTION

On the surface of the Earth, solar power is relatively low in the density and its illumination is intermittent. Light‐harvesting systems are required for the efficient transformation of the sunlight energy into the other usable energies, including chemical and electrical energies. In phototrophs, photosynthetic antennas are utilized for effectively absorbing and harvesting solar light. A variety of pigments are contained in the light‐harvesting antenna systems,^1^, ^2^ where chlorophylls (Chls) are major pigments.^3^, ^4^ A large number of the pigment molecules generally interact with peptides to form such photosynthetic antennas.^5^, ^6^, ^7^, ^8^, ^9^, ^10^, ^11^, ^12^, ^13^ In some photosynthetic bacteria, specific Chl molecules self‐aggregate without any assistance of peptides to produce the core of their antennas, which are called chlorosomes.^14^, ^15^, ^16^, ^17^ The chlorosomal self‐aggregates were mimicked so far, and their model systems have been already reported.^17^, ^18^, ^19^, ^20^, ^21^, ^22^, ^23^

**FIGURE 1:**
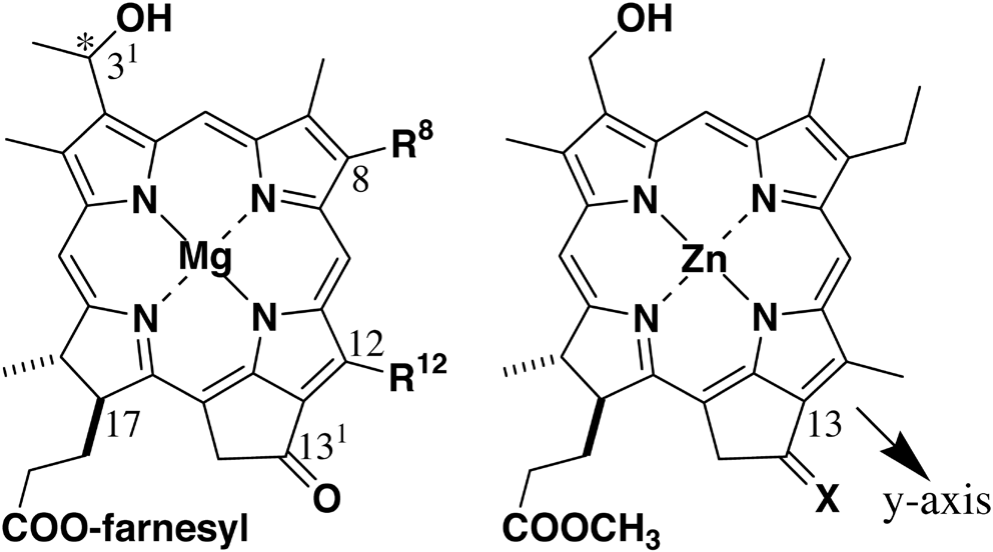
Molecular structures of natural BChl‐*d* (left) and its synthetic models (right): R^8^=CH_2_CH_3_, CH_2_CH_2_CH_3_, CH_2_CH(CH_3_)_2_, CH_2_C(CH_3_)_3_, R^12^=CH_3_, CH_2_CH_3_, X=O, NOH, NOR″, NOCOR′, NNHCOR.

Bacteriochlorophyll(BChl)‐*d* (see the left drawing in Figure 1) is one of the chlorosomal Chl pigments. A zinc‐metalated BChl‐*d* analog lacking the 3^1^‐methyl group and simplifying the farnesyl group in the 17‐propionate residue to a methyl group (X=O in Figure 1, right) was reported to be a good model for the preparation of the chlorosome‐like self‐aggregates.^24^ The synthetic model self‐aggregates in a hydrophobic environment to give largely red‐shifted and broadened absorption bands in visible and near‐infrared regions, compared with its monomeric bands in a polar organic solvent,^24^, ^25^ similar to the BChl‐*d* self‐aggregates in chlorosomes. Recently, we described that the 13‐oxime (X=NOH) and its alkylated and acylated derivatives (X=NOR″ and NOCOR′) were prepared and self‐aggregated inside an aqueous Triton X‐100 (TX‐100) micelle as one of the hydrophobic spheres to give chlorosome‐like self‐aggregates.^25^, ^26^, ^27^

The 13^1^‐oxo group in some Chl molecules has been already substituted with =N–N moieties.^28^, ^29^, ^30^, ^31^, ^32^, ^33^, ^34^, ^35^ Here, we report on the synthesis of novel BChl‐*d* model pigments possessing an acylhydrazone moiety (X=NNHCOR in Figure 1, right) and their self‐aggregation in an aqueous TX‐100 solution. The chlorosome‐like self‐aggregation was analyzed by ultraviolet (UV)–visible absorption and circular dichroism (CD) spectroscopy and found to be dependent on the R‐substituents.

## MATERIALS AND METHODS

### Apparatus

UV–visible absorption and CD spectra were measured with a Hitachi U‐3500/4100 spectrometer and a JASCO J‐720W/1500 spectrometer, respectively. ^1^H NMR spectra were measured at room temperature with a JEOL ECA‐600 (600 MHz) or AL400 (400 MHz) spectrometer; chemical shifts (δs) are expressed in parts per million relative to tetramethylsilane (δ = 0.00 ppm) as an internal reference. Proton peaks were assigned using ^1^H–^1^H two‐dimensional NMR techniques. High‐resolution mass spectra (HRMS) were recorded on a Bruker micrOTOF II spectrometer: electrospray ionization (ESI) and positive mode in methanol. Flash column chromatography (FCC) was carried out with silica gel (Merck Kieselgel 60, 0.040–0.063 mm). High‐performance liquid chromatography (HPLC) was performed on a packed octadecylated column (Inertsil ODS‐P or ODS‐EP 5 μm, 10 mmϕ × 250 mm; GL Sciences) with a Shimadzu LC‐20AT pump and SPD‐M10A photodiode‐array detector or LC‐20AR pumps and SPD‐M40 photodiode‐array detector.

### Materials

**SCHEME 1:**
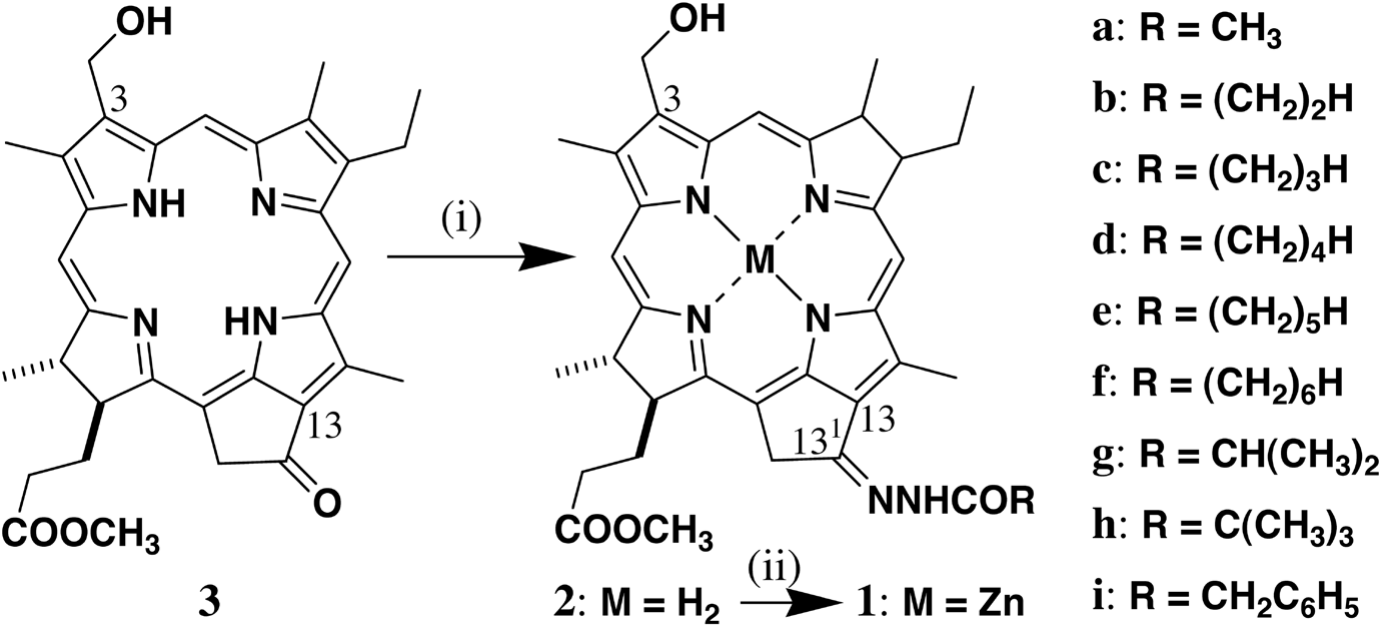
Synthesis of zinc 3‐hydroxymethyl‐pyropheophorbides‐*a* **1a**–**i** possessing an *N*‐acylhydrazone moiety at the 13‐position: (i) H_2_NNHCOR/MeOH, AcOH; (ii) Zn(OAc)_2_·2H_2_O/MeOH, CH_2_Cl_2_.

Methyl 3‐devinyl‐3‐hydroxymethyl‐pyropheophorbide‐*a* (**3**, see the left drawing in Scheme 1) was prepared according to reported procedures.^24^ All the reaction reagents and solvents were obtained from commercial suppliers and utilized as supplied. TX‐100 was purchased from Nacalai Tesque and used as received. For optical spectroscopy, tetrahydrofuran (THF) and distilled water were purchased from Nacalai Tesque as reagents prepared specially for HPLC.

### Synthesis of bacteriopheophorbide‐*d* analogs **2a–i**

13^1^‐Oxo‐chlorin **3** (22.1 mg, 40 μmol) was dissolved in methanol (30 mL) in the presence of acetic acid (2 mL), to which was added commercially available acylhydrazine (RCONHNH_2_, 2 mmol, 50 eq.) at room temperature in the dark under nitrogen. The solution was refluxed for 15 h and cooled down to room temperature. The reaction mixture was diluted with dichloromethane (50 mL), washed with an aqueous 4% sodium hydrogen carbonate solution and distilled water, dried over sodium sulfate, and filtered. All the solvents were evaporated, and the residue was purified by FCC with dichloromethane and 2% methanol and precipitation from dichloromethane and excess hexane to give the corresponding acylhydrazones **2a** (R=methyl, 81% isolated yield), **2b** (ethyl, 51%), **2c** (propyl, 51%), **2d** (butyl, 41%), **2e** (pentyl, 49%), **2f** (hexyl, 53%), **2g** (isopropyl, 63%), **2h** (*tert*‐butyl, 82%), and **2i** (benzyl, 69%). All their spectral data are available in Supporting Information.

### Synthesis of Zn‐BChl‐*d* analogs **1a–i**

Free base **2** (10 μmol) was dissolved into dichloromethane (5 mL) in the dark under nitrogen at room temperature, to which a methanol solution (2 mL) saturated with zinc acetate dihydrate (excess) was added. After stirred for 10 h, the reaction mixture was diluted with dichloromethane (20 mL), washed with an aqueous solution saturated with sodium hydrogen carbonate and distilled water, dried over sodium sulfate, and filtered. All the solvents were evaporated, and the residue was purified by reverse‐phase (RP) HPLC with methanol and 10% water to give the corresponding zinc complexes **1a** (R=methyl, 12% isolated yield), **1b** (ethyl, 17%), **1c** (propyl, 16%), **1d** (butyl, 36%), **1e** (pentyl, 14%), **1f** (hexyl, 18%), **1g** (isopropyl, 50%), **1h** (*tert*‐butyl, 30%), and **1i** (benzyl, 43%). All their spectral data are available in Supporting Information.

## RESULTS AND DISCUSSION

### Synthesis of Zn‐BChl‐*d* analogs with *N*‐acylhydrazone

**FIGURE 2:**
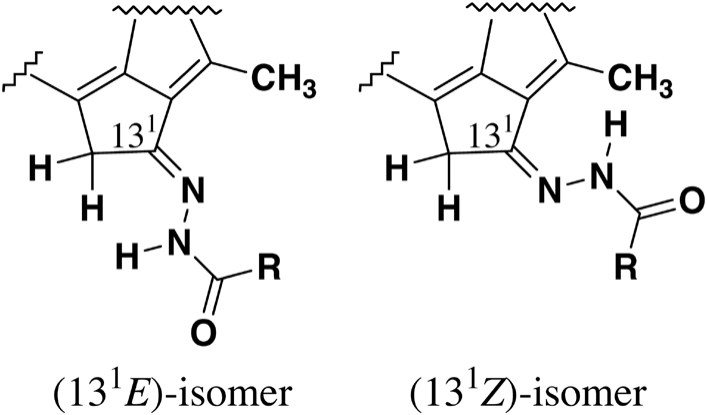
Configurations around the 13^1^‐substituents in hydrazones **1** and **2**.

Methyl 3‐hydroxymethyl‐pyropheophorbide‐*a* (**3**) as the 3^1^‐demethyl form of a bacteriopheophorbide‐*d* homolog possessing ethyl and methyl groups at the 8‐ and 12‐positions, respectively, was prepared from the chemical modification of naturally occurring chlorophyll‐*a*.^24^ The 13‐carbonyl group in **3** was reacted with acetylhydrazine in the presence of acetic acid in methanol [step (i) in Scheme 1]^28^, ^31^, ^36^, ^37^ to give the corresponding hydrazone **2a** (R=CH_3_ in Scheme 1, right) after stirring at room temperature for 15 h. The condensation reaction was so clean that the isolated yield was 81%. The other acylhydrazines with straight and branched alkyl chains as well as a benzyl group were dehydratively condensed with **3** to afford **2b**–**i** (Scheme 1, right). The ^1^H NMR spectra of the products in deuterated chloroform exhibited that they were single configurations of the 13^1^‐acylhydrazinylidene groups. The nuclear Overhauser effect spectroscopy (NOESY) of **2f** (Figure S3, upper) indicated that it was the (13^1^ *E*)‐isomer (Figure 2, left). Since the chemical shifts of the protons near the 13^1^‐position of **2a**–**e**/**g**–**i** were comparable to those of **2f** (see the data in Supporting Information), all the hydrazones were proposed to be the (13^1^ *E*)‐isomers.

The treatment of free bases **2a**–**i** in dichloromethane with a methanol solution saturated with zinc acetate dihydrate at room temperature [step (ii) in Scheme 1] afforded the corresponding zinc complexes as a mixture of isomeric products, except for a single product obtained from **2h** bearing the pivaloyl group. After the separation of the resulting products by RP‐HPLC, the major products as the second fractions were fully characterized by visible absorption and ^1^H NMR spectroscopies as well as mass spectrometry. One‐ and/or two‐dimensional ^1^H NMR spectra of the major products, **1a**–**i**, indicated that they were (13^1^ *E*)‐stereoisomers (Figure 2, left and Figure S3, lower). The molecular structures of the minor products as the first fractions could not be determined from ^1^H NMR spectral analyses but were proposed to be (13^1^ *Z*)‐isomers (Figure 2, right). It is noted that the rotational isomerization around the C13^1^=N imine bond would occur during the aforementioned zinc metalation procedures, which is consistent with the stereochemical conversion observed for the related acyloximes (X=NOCOR′ in Figure 1, right).^27^ Molecular modeling of these isomers supported that the (13^1^ *E*)‐isomers were more energetically stable than the (13^1^ *Z*)‐isomers (Table S1), so the former isomers were obtained more largely than the latter ones. The calculation proved that the *cis*‐conformation around the present amide bond shown in Figure 2 was preferable over the *trans* (Table S2). Especially, the sterically demanding *tert*‐butyl group in **1**/**2h** [R=C(CH_3_)_3_ in Figure 2, left] disturbed the attack of a proton or zinc ion toward the imino nitrogen at the 13^1^‐position, which induced the aforementioned isomerization of the (13^1^ *E*)‐ to (13^1^ *Z*)‐configuration.

### Self‐aggregation of Zn‐BChl‐*d* analogs with *N*‐acylhydrazone

**FIGURE 3:**
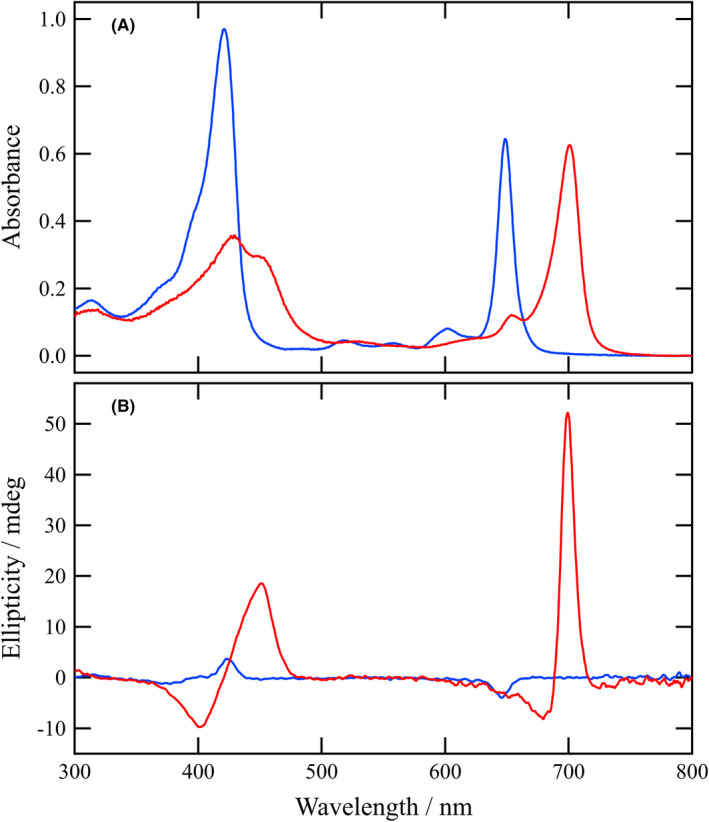
UV–visible absorption (A) and CD spectra (B) of **1a** (10 μM) in THF (blue line) and in an aqueous 0.025% (wt/v) TX‐100 solution containing 1% (v/v) THF (red line).

Acetylhydrazone **1a** was well‐dissolved into THF to give sharp and intense visible absorption bands at 649 and 421 nm, which are called Qy and Soret bands, respectively (the blue line in Figure 3a). The spectral feature indicated that **1a** was dispersed as a monomeric species in THF. The oxygen atom of a THF molecule coordinated to the central zinc atom of **1a**, and the axially coordinated species could not interact intramolecularly at a low concentration (ca. 10 μM). In the Qy and Soret regions, weak negative and positive CD bands appeared (the blue line in Figure 3B), supporting that **1a** was monomeric in a diluted THF solution. All the other acylhydrazones **1b**–**i** exhibited almost the same UV–visible absorption spectra in THF as that of **1a**. No substitution effect on the monomeric bands was observed by changes of the terminal alkyl groups in the 13^1^‐substituents. Therefore, acylhydrazones **1a**–**i** possessed almost the identical electronic states to their core chlorin π‐systems.

A concentrated THF solution of **1a** (1 mM) was mixed with nonionic detergent TX‐100 [2.5% (wt/v)] and diluted with 99‐fold distilled water at room temperature. The aqueous micellar solution exhibited a different UV–visible absorption spectrum (the red line in Figure 3a) from that in neat THF (blue line). New bands appeared at 701 and 449 nm, which were red‐shifted from the monomeric bands. The shift values were estimated to be 1150 and 1490 cm^−1^, respectively. The spectral change was similar to those by self‐aggregation in natural chlorosomes and their model systems,^17^ although the red‐shift value of Qy maxima in **1a** was smaller than those in 13‐ketone (1470 cm^−1^; X=O in Figure 1, right)^25^ and acetyloxime (1360 cm^−1^; X=NOCOCH_3_ in Figure 1, right).^27^ Both the new bands were broader than the corresponding monomeric bands, supporting that **1a** self‐aggregated in a chlorosomal aggregation manner. At the red‐shifted peak positions, intense positive CD bands were observed (the red line in Figure 3b) and accompanied the negative bands on the blue sides. The enhanced CD spectrum in the aqueous micellar solution in comparison with the small bands in the monomeric THF solution showed that a large number of **1a** molecules stacked with a slipped face of the chlorin π‐planes to give exciton‐coupled J‐aggregates inside an aqueous micelle. The situation also supported that **1a** produced chlorosome‐like self‐aggregates in the hydrophobic environment.

**FIGURE 4:**
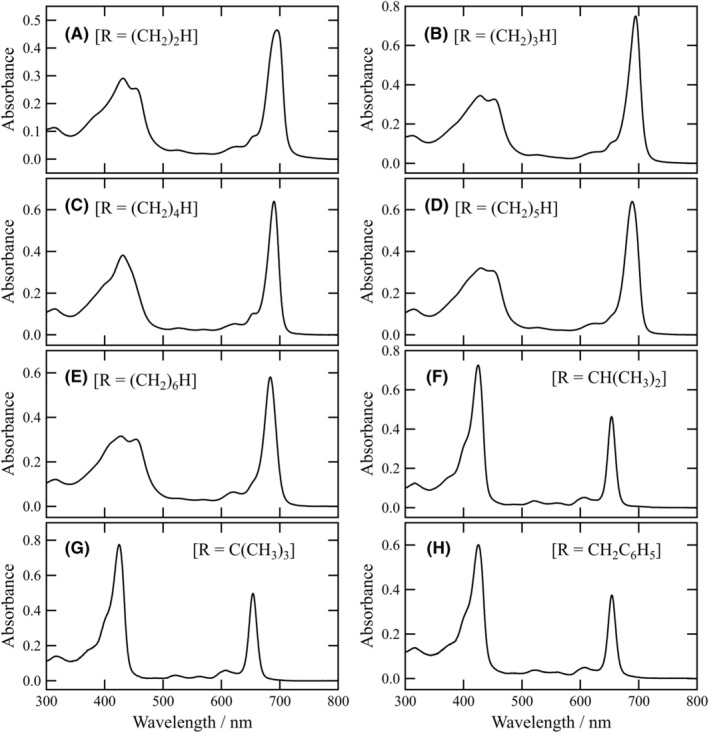
UV–visible absorption spectra of **1b**–**i** (10 μM) in an aqueous 0.025% (wt/v) TX‐100 solution containing 1% (v/v) THF: **1b** (A), **1c** (B), **1d** (C), **1e** (D), **1f** (E), **1g** (F), **1h** (G), and **1i** (H).

In acylhydrazones **1b**–**f** possessing a straight alkyl chain at the acyl group, similar UV–visible absorption spectra as in **1a** were observed in an aqueous TX‐100 solution (Figure 4A–E). The red‐shifted Qy maxima were dependent on the length of the oligomethylene moiety. The elongation moved the Qy maxima to lower wavelengths: 701 (**1a**) > ≈695 (**1b**/**c**) > ≈690 (**1d**/**e**) > 684 nm (**1f**). The decrease in the red‐shift values was ascribable to the suppression of the self‐aggregation with an increase in the number from one to six in (CH_2_)_n_H of the terminal alkyl group. The increase in the steric factor of the alkyl chains partially disturbed the self‐aggregation. The Qy maximum of **1b** (*n* = 2) was almost the same as that of **1c** (*n* = 3), and that of **1d** (*n* = 4) was comparable to that of **1e** (*n* = 5). The consistency may be due to an odd–even effect which is often observed in supramolecular aggregation.^38^

**FIGURE 5:**
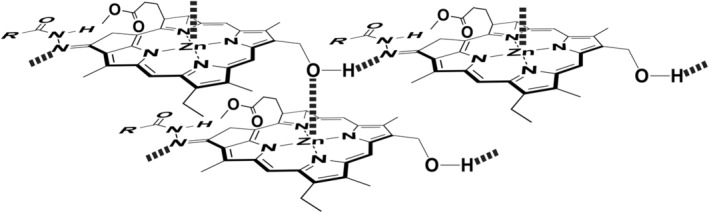
Proposed supramolecular structure of self‐aggregate of **1**.

For isopropyl **1g**, *tert*‐butyl **1h**, and benzyl substitutes **1i**, no red‐shifted absorption bands were measured in an aqueous solution, and monomeric bands were visible at 654 and 425 nm (Figure 1F–H). No self‐aggregation in **1g**–**i** was ascribed to the steric hindrance of their terminal substituents. The steric effect of the acyl groups in **1a**–**i** on their self‐aggregation indicated that the 13^1^‐imino nitrogen atom was important for the production of the self‐aggregates. Based on the previous reports,^25^, ^26^, ^27^ the 3^1^‐hydroxy group of a **1a**–**f** molecule coordinated to the central zinc atom of another molecule, and the coordinated OH group hydrogen‐bonded to the 13^1^‐imino group of the third molecule. The specific interaction of Zn^…^O3^2^–H^…^N=C13^1^ led to the π–π stacking of chlorin chromophores along the molecular *y*‐axis (see the arrow in Figure 1, right) to give chlorosome‐like self‐aggregates as shown in Figure 5.

## CONCLUSION

Zn‐BChl‐*d* analogs **1a**–**i** possessing an acylhydrazone moiety as the 13‐keto‐carbonyl alternative were prepared through the simple condensation of the 13^1^‐oxo group in **3** with a variety of acylhydrazines. Synthetic **1a**–**f** bearing straight alkyl chains in the acyl substitute self‐aggregated in an aqueous TX‐100 solution to give oligomers with red‐shifted and broadened Qy and Soret absorption bands and intense CD bands in the red‐shifted regions. The spectral changes from the monomeric species were ascribable to the formation of chlorosome‐like exciton‐coupled J‐aggregates by the specific Zn^…^O3^2^–H^…^N=C13^1^ bond and π–π interaction of the composite chlorin chromophore along the molecular *y*‐axis. The steric effect around the 13^1^‐imino nitrogen atom requisite for the above hydrogen‐bonding regulated the chlorosome‐like self‐aggregation in an aqueous micelle. The amide groups attached to the 13^1^=N atom were situated in the *E*‐configuration and took the *cis*‐conformation, so the terminal substituents R in the 13‐acylhydrazone moiety were close to the 13^1^=N. An increase in the steric hindrance of the R‐substituents (methyl to hexyl) moved the red‐shifted Qy maxima to shorter wavelengths and fully suppressed the self‐aggregation of **1g**–**i** bearing isopropyl, *tert*‐butyl, and benzyl as the R. Readily available acylhydrazones **1** will provide a new class of BChl‐*d* analogs to give chlorosome‐like self‐aggregates with tunable optical properties.

## Supporting information


Data S1
