# Supplementary material for: Self‐aggregation of zinc bacteriochlorophyll‐d analogs with an acylhydrazone moiety as the 13‐keto‐carbonyl alternative
Source: Photochem Photobiol. 2024 Apr 6;101(1):63–9. doi: 10.1111/php.13949 (PMC11737012; doi:10.1111/php.13949)
Supplement: Supplementary file 1 — Data S1 [file PHP-101-63-s001.docx]

**Supporting Information**

**Self-aggregation of zinc bacteriochlorophyll-*d* analogs with an acylhydrazone moiety as the 13-keto-carbonyl alternative**

Satoru Fujii and Hitoshi Tamiaki*

*Graduate School of Life Sciences, Ritsumeikan University, Kusatsu, Shiga 525-8577, Japan*

* Corresponding author email: tamiaki@fc.ritsumei.ac.jp (H. Tamiaki)

**Table of contents**

Synthesis of **2a–i**/**1a–i** with their ^1^H NMR spectra (**Figs. S1/S2**)............................S2–S19

Calculated energy of (13^1^*E*/*Z*)-**1** and **2** (**Table S1**).........................................................S20

Calculated energy of *cis*/*trans*-conformers in (13^1^*E*)-**1** and **2** (**Table S2**).......................S21

^1^H–^1^H NOESY of **2f**/**1f** (**Fig. S3**)...................................................................................S22

**Synthesis of bacteriopheophorbide-*d* analogs 2a–i.**

Methyl (131*E*)-acetylhydrazinylidene-131-deoxo-3-devinyl-3-hydroxymethyl-pyropheophorbide-*a* (**2a**): Condensation of **3** with acetylhydrazine (148 mg) gave the titled hydrazone (19.7 mg, 32.4 μmol, 81%): dark green solid; mp = 211 °C; Vis (CH_2_Cl_2_) λ_max_/nm = 668 (relative intensity 0.64), 609 (0.05), 505 (0.12), 407 (1.00); ^1^H NMR (600 MHz, CDCl_3_) δ/ppm = 9.75 (1H, s, 5-H), 9.56 (1H, s, 10-H), 8.88 (1H, s, 13^1^=NNH), 8.79 (1H, s, 20-H), 6.01 (2H, s, 3-CH_2_), 5.19, 5.05 (each 1H, d, *J* = 18 Hz, 13^1^-CH_2_), 4.54 (1H, dq, *J* = 2, 7.5 Hz, 18-H), 4.30 (1H, dt, *J* = 9, 2 Hz, 17-H), 3.79 (2H, q, *J* = 8 Hz, 8-CH_2_), 3.60 (3H, s, 12-CH_3_), 3.53 (3H, s, 17^2^-COOCH_3_), 3.52 (3H, s, 2-CH_3_), 3.37 (3H, s, 7-CH_3_), 2.64–2.54, 2.25–2.20, 2.17–2.11 (2H+1H+1H, m, 17-CH_2_CH_2_), 2.61 (3H, s, NCOCH_3_), ≈2.3 (1H, br, 3^1^-OH), 1.81 (1H, d, *J* = 7.5 Hz, 18-CH_3_), 1.74 (3H, t, *J* = 8 Hz, 8^1^-CH_3_), −0.91, −2.86 (each 1H, br-s, NH×2); HRMS (ESI) found: *m*/*z* = 609.3184. calcd for C_35_H_41_N_6_O_4_: [M+H]^+^, 609.3184.

**Figure S1a.** ^1^H NMR spectrum (600 MHz) of **2a** in CDCl_3_.

Methyl 131-deoxo-3-devinyl-3-hydroxymethyl-(131*E*)-propanoylhydrazinylidene-pyropheophorbide-*a* (**2b**): Condensation of **3** with propanoylhydrazine (propionyl-hydrazine, 176 mg) gave the titled hydrazone (12.7 mg, 20.4 μmol, 51%): dark green solid; mp = 168 °C; Vis (CH_2_Cl_2_) λ_max_/nm = 668 (relative intensity 0.63), 610 (0.05), 505 (0.12), 407 (1.00); ^1^H NMR (600 MHz, CDCl_3_) δ/ppm = 9.73 (1H, s, 5-H), 9.54 (1H, s, 10-H), 8.87 (1H, s, 13^1^=NNH), 8.77 (1H, s, 20-H), 5.99 (2H, s, 3-CH_2_), 5.16, 5.04 (each 1H, d, *J* = 18 Hz, 13^1^-CH_2_), 4.53 (1H, q, *J* = 7 Hz, 18-H), 4.28 (1H, dd, *J* = 10, 1 Hz, 17-H), 3.78 (2H, q, *J* = 7.5 Hz, 8-CH_2_), 3.59 (3H, s, 12-CH_3_), 3.50 (6H, s, 2-CH_3_, 17^2^-COOCH_3_), 3.36 (3H, s, 7-CH_3_), 3.04, 3.00 (each 1H, dq, *J* = 16, 8 Hz, NCOCH_2_), 2.61–2.52, 2.23–2.09 (each 2H, m, 17-CH_2_CH_2_), 2.32 (1H, br, 3^1^-OH), 1.80 (3H, d, *J* = 7 Hz, 18-CH_3_), 1.73 (3H, t, *J* = 7.5 Hz, 8^1^-CH_3_), 1.44 (3H, t, *J* = 8 Hz, NCOCCH_3_), −0.94, −2.88 (each 1H, br-s, NH×2); HRMS (ESI) found: *m*/*z* = 623.3340, calcd for C_36_H_43_N_6_O_4_: [M+H]^+^, 623.3340.

**Figure S1b.** ^1^H NMR spectrum (600 MHz) of **2b** in CDCl_3_.

Methyl (131*E*)-butanoylhydrazinylidene-131-deoxo-3-devinyl-3-hydroxymethyl-pyropheophorbide-*a* (**2c**): Condensation of **3** with butanoylhydrazine (butyrylhydrazine, 204 mg) gave the titled hydrazone (13.0 mg, 20.4 μmol, 51%): dark green solid; mp = 178 °C; Vis (CH_2_Cl_2_) λ_max_/nm = 668 (relative intensity 0.62), 610 (0.05), 505 (0.12), 408 (1.00); ^1^H NMR (600 MHz, CDCl_3_) δ/ppm = 9.77 (1H, s, 5-H), 9.62 (1H, s, 10-H), 8.99 (1H, s, 13^1^=NNH), 8.81 (1H, s, 20-H), 6.03 (2H, s, 3-CH_2_), 5.29, 5.17 (each 1H, d, *J* = 18 Hz, 13^1^-CH_2_), 4.58 (1H, dq, *J* = 2, 7 Hz, 18-H), 4.34 (1H, dt, *J* = 9, 2 Hz, 17-H), 3.82 (2H, q, *J* = 8 Hz, 8-CH_2_), 3.621 (3H, s, 12-CH_3_), 3.617 (3H, s, 17^2^-COOCH_3_), 3.53 (3H, s, 2-CH_3_), 3.39 (3H, s, 7-CH_3_), 3.04. 3.01 (each 1H, dt, *J* = 15, 7.5 Hz, NCOCH_2_), 2.69–2.57, 2.30–2.24, 2.21–2.14 (2H+1H+1H, m, 17-CH_2_CH_2_), ≈2.3 (1H, br, 3^1^-OH), 1.99 (2H, sextet, *J* = 7.5 Hz, NCOCCH_2_), 1.81 (3H, d, *J* = 7 Hz, 18-CH_3_), 1.76 (3H, t, *J* = 8 Hz, 8^1^-CH_3_), 1.22 (3H, t, *J* = 7.5 Hz, NCOC_2_CH_3_), −0.85, −2.84 (each 1H, br-s, NH×2); HRMS (ESI) found: *m*/*z* = 637.3498, calcd for C_37_H_45_N_6_O_4_: [M+H]^+^, 637.3497.

**Figure S1c.** ^1^H NMR spectrum (600 MHz) of **2c** in CDCl_3_.

Methyl 131-deoxo-3-devinyl-3-hydroxymethyl-(131*E*)-pentanoylhydrazinylidene-pyropheophorbide-*a* (**2d**): Condensation of **3** with pentanoylhydrazine (valerylhydrazine, 232 mg) gave the titled hydrazone (10.7 mg, 16.4 μmol, 41%): dark green solid; mp = 176 °C; Vis (CH_2_Cl_2_) λ_max_/nm = 668 (relative intensity 0.66), 610 (0.04), 505 (0.12), 406 (1.00); ^1^H NMR (600 MHz, CDCl_3_) δ/ppm = 9.76 (1H, s, 5-H), 9.61 (1H, s, 10-H), 8.86 (1H, s, 13^1^=NNH), 8.80 (1H, s, 20-H), 6.01 (2H, s, 3-CH_2_), 5.29, 5.16 (each 1H, d, *J* = 17.5 Hz, 13^1^-CH_2_), 4.58 (1H, br-q, *J* = 7 Hz, 18-H), 4.35 (1H, br-d, *J* = 7 Hz, 17-H), 3.80 (2H, q, *J* = 8 Hz, 8-CH_2_), 3.61 (3H, s, 12-CH_3_), 3.59 (3H, s, 17^2^-COOCH_3_), 3.52 (3H, s, 2-CH_3_), 3.37 (3H, s, 7-CH_3_), 3.05, 3.02 (each 1H, dt, *J* = 14.5, 7 Hz, NCOCH_2_), 2.69–2.56, 2.29–2.17 (each 2H, m, 17-CH_2_CH_2_), ≈2.3 (1H, br, 3^1^-OH), 1.94 (2H, quintet, *J* = 7 Hz, NCOCCH_2_), 1.82 (3H, d, *J* = 7 Hz, 18-CH_3_), 1.74 (3H, t, *J* = 8 Hz, 8^1^-CH_3_), 1.62 (2H, sextet, *J* = 7.5 Hz, NCOC_2_CH_2_), 1.10 (3H, t, *J* = 7.5 Hz, NCOC_3_CH_3_), −0.84, −2.83 (each 1H, br-s, NH×2); HRMS (ESI) found: *m*/*z* = 651.3654, calcd for C_38_H_47_N_6_O_4_: [M+H]^+^, 651.3653.

**Figure S1d.** ^1^H NMR spectrum (600 MHz) of **2d** in CDCl_3_.

Methyl 131-deoxo-3-devinyl-(131*E*)-hexanoylhydrazinylidene-3-hydroxymethyl-pyropheophorbide-*a* (**2e**): Condensation of **3** with hexanoylhydrazine (caproylhydrazine, 260 mg) gave the titled hydrazone (13.0 mg, 19.6 μmol, 49%): dark green solid; mp = 162 °C; Vis (CH_2_Cl_2_) λ_max_/nm = 668 (relative intensity 0.65), 609 (0.04), 505 (0.12), 406 (1.00); ^1^H NMR (600 MHz, CDCl_3_) δ/ppm = 9.75 (1H, s, 5-H), 9.60 (1H, s, 10-H), 8.83 (1H, s, 13^1^=NNH), 8.79 (1H, s, 20-H), 6.01 (2H, s, 3-CH_2_), 5.26, 5.14 (each 1H, d, *J* = 18 Hz, 13^1^-CH_2_), 4.57 (1H, br-q, *J* = 7 Hz, 18-H), 4.33 (1H, br-d, *J* = 9 Hz, 17-H), 3.80 (2H, q, *J* = 7.5 Hz, 8-CH_2_), 3.59 (6H, s, 12-CH_3_, 17^2^-COOCH_3_), 3.52 (3H, s, 2-CH_3_), 3.37 (3H, s, 7-CH_3_), 3.03, 3.00 (each 1H, dt, *J* = 14.5, 7.5 Hz, NCOCH_2_), 2.66–2.53, 2.24–2.18 (each 2H, m, 17-CH_2_CH_2_), ≈2.3 (1H, br, 3^1^-OH), 1.95 (2H, quintet, *J* = 7.5 Hz, NCOCCH_2_), 1.81 (3H, d, *J* = 7.5 Hz, 18-CH_3_), 1.74 (3H, t, *J* = 7.5 Hz, 8^1^-CH_3_), 1.60–1.54 (2H, m, NCOC_2_CH_2_), 1.51 (2H, sextet, *J* = 7 Hz, NCOC_3_CH_2_), 1.02 (3H, t, *J* = 7 Hz, NCOC_4_CH_3_), −0.87, −2.85 (1H, br-s, NH×2); HRMS (ESI) found: *m*/*z* = 665.3805, calcd for C_39_H_49_N_6_O_4_: [M+H]^+^, 665.3810.

**Figure S1e.** ^1^H NMR spectrum (600 MHz) of **2e** in CDCl_3_.

Methyl 131-deoxo-3-devinyl-(131*E*)-heptanoylhydrazinylidene-3-hydroxymethyl-pyropheophorbide-*a* (**2f**): Condensation of **3** with heptanoylhydrazine (enanthyl-hydrazine, 288 mg) gave the titled hydrazone (14.4 mg, 21.2 μmol, 53%): dark green solid; mp = 160 °C; Vis (CH_2_Cl_2_) λ_max_/nm = 668 (relative intensity 0.66), 609 (0.06), 505 (0.13), 407 (1.00); ^1^H NMR (600 MHz, CDCl_3_) δ/ppm = 9.77 (1H, s, 5-H), 9.62 (1H, s, 10-H), 8.93 (1H, s, 13^1^=NNH), 8.80 (1H, s, 20-H), 6.03 (2H, s, 3-CH_2_), 5.30, 5.18 (each 1H, d, *J* = 18 Hz, 13^1^-CH_2_), 4.58 (1H, dq, *J* = 2, 7 Hz, 18-H), 4.34 (1H, dt, *J* = 9.5, 2 Hz, 17-H), 3.81 (2H, q, *J* = 8 Hz, 8-CH_2_), 3.62 (3H, s, 12-CH_3_), 3.61 (3H, s, 17^2^-COOCH_3_), 3.53 (3H, s, 2-CH_3_), 3.38 (3H, s, 7-CH_3_), 3.05, 3.01 (each 1H, dt, *J* = 14.5, 7.5 Hz, NCOCH_2_), 2.69–2.58, 2.29–2.16 (each 2H, m, 17-CH_2_CH_2_), ≈2.3 (1H, br, 3^1^-OH), 1.94 (2H, quintet, *J* = 7.5 Hz, NCOCCH_2_), 1.81 (3H, d, *J* = 7 Hz, 18-CH_3_), 1.75 (3H, t, *J* = 8 Hz, 8^1^-CH_3_), 1.59 (2H, quintet, *J* = 7.5 Hz, NCOC_2_CH_2_), 1.50–1.40 (4H, m, NCOC_3_CH_2_CH_2_), 0.97 (3H, t, *J* = 7 Hz, NCOC_5_CH_3_), −0.84, −2.84 (each 1H, br-s, NH×2); HRMS (ESI) found: *m*/*z* = 679.3954, calcd for C_40_H_51_N_6_O_4_: [M+H]^+^, 679.3966.

**Figure S1f.** ^1^H NMR spectrum (600 MHz) of **2f** in CDCl_3_.

Methyl 131-deoxo-3-devinyl-3-hydroxymethyl-(131*E*)-(2-methylpropanoyl)-hydrazinylidene-pyropheophorbide-*a* (**2g**): Condensation of **3** with (2-methylpropanoyl)-hydrazine (isobutyrylhydrazine, 204 mg) gave the titled hydrazone (16.0 mg, 25.1 μmol, 63%): dark green solid; mp = 202 °C; Vis (CH_2_Cl_2_) λ_max_/nm = 668 (relative intensity 0.62), 610 (0.05), 505 (0.12), 408 (1.00); ^1^H NMR (600 MHz, CDCl_3_) δ/ppm = 9.71 (1H, s, 5-H), 9.60 (1H, s, 10-H), 8.98 (1H, s, 13^1^=NNH), 8.82 (1H, s, 20-H), 5.91 (2H, s, 3-CH_2_), 5.35, 5.23 (each 1H, d, *J* = 17 Hz, 13^1^-CH_2_), 4.59 (1H, br-q, *J* = 7 Hz, 18-H), 4.36 (1H, br-d, *J* = 7 Hz, 17-H), 3.83 (1H, septet, *J* = 7 Hz, NCOCH), 3.76 (2H, q, *J* = 8 Hz, 8-CH_2_), 3.62 (3H, s, 12-CH_3_), 3.58 (3H, s, 17^2^-COOCH_3_), 3.47 (3H, s, 2-CH_3_), 3.32 (3H, s, 7-CH_3_), 2.70–2.54, 2.29–2.20 (each 2H, m, 17-CH_2_CH_2_), ≈2.3 (1H, br, 3^1^-OH), 1.83 (1H, d, *J* = 7 Hz, 18-CH_3_), 1.70 (3H, t, *J* = 8 Hz, 8^1^-CH_3_), 1.47, 1.45 (each 3H, d, *J* = 7 Hz, NCOC(CH_3_)_2_), −1.01, −2.91 (each 1H, br-s, NH×2); HRMS (ESI) found: *m*/*z* = 634.3499, calcd for C_37_H_45_N_6_O_4_: [M+H]^+^, 637.3497.

**Figure S1g.** ^1^H NMR spectrum (600 MHz) of **2g** in CDCl_3_.

Methyl 131-deoxo-3-devinyl-(131*E*)-(2,2-dimethylpropanoyl)hydrazinylidene-3-hydroxymethyl-pyropheophorbide-*a* (**2h**): Condensation of **3** with (2,2-dimethyl-propanoyl)hydrazine (pivaloylhydrazine, 232 mg) gave the titled hydrazone (21.3 mg, 32.7 μmol, 82%): dark green solid; mp = 156 °C; Vis (CH_2_Cl_2_) λ_max_/nm = 668 (relative intensity 0.65), 610 (0.05), 505 (0.12), 408 (1.00); ^1^H NMR (600 MHz, CDCl_3_) δ/ppm = 9.72 (1H, s, 5-H), 9.59 (1H, s, 10-H), 9.11 (1H, s, 13^1^=NNH), 8.77 (1H, s, 20-H), 5.99 (2H, s, 3-CH_2_), 5.57, 5.35 (each 1H, d, *J* = 17.5 Hz, 13^1^-CH_2_), 4.56 (1H, q, *J* = 7 Hz, 18-H), 4.34 (1H, d, *J* = 10 Hz, 17-H), 3.76 (2H, q, *J* = 7 Hz, 8-CH_2_), 3.73 (3H, s, 12-CH_3_), 3.68 (3H, s, 17^2^-COOCH_3_), 3.50 (3H, s, 2-CH_3_), 3.35 (3H, s, 7-CH_3_), 2.77–2.66, 2.38–2.11 (each 2H, m, 17-CH_2_CH_2_), 2.23 (1H, br, 3^1^-OH), 1.80 (3H, d, *J* = 7 Hz, 18-CH_3_), 1.72 (3H, t, *J* = 7 Hz, 8^1^-CH_3_), 1.57 (9H, s, NCOC(CH_3_)_3_), −0.74, −2.77 (each 1H, br-s, NH×2); HRMS (ESI) found: *m*/*z* = 651.3658, calcd for C_38_H_47_N_6_O_4_: [M+H]^+^, 651.3653.

**Figure S1h.** ^1^H NMR spectrum (600 MHz) of **2h** in CDCl_3_.

Methyl 131-deoxo-3-devinyl-3-hydroxymethyl-(131*E*)-(phenylacetyl)-hydrazinylidene-pyropheophorbide-*a* (**2i**): Condensation of **3** with (phenylacetyl)-hydrazine (300 mg) gave the titled hydrazone (18.9 mg, 27.6 μmol, 69%): dark green solid; mp = 156 °C; Vis (CH_2_Cl_2_) λ_max_/nm = 668 (relative intensity 0.66), 610 (0.05), 505 (0.13), 407 (1.00); ^1^H NMR (600 MHz, CDCl_3_) δ/ppm = 9.76 (1H, s, 5-H), 9.65 (1H, s, 10-H), 8.96 (1H, s, 13^1^=NNH), 8.80 (1H, s, 20-H), 7.63 (2H, d, *J* = 8 Hz, *o*-H of Ph), 7.41 (2H, t, *J* = 8 Hz, *m*-H of Ph), 7.32 (1H, t, *J* = 8 Hz, *p*-H of Ph), 6.03 (2H, s, 3-CH_2_), 5.37, 5.24 (each 1H, d, *J* = 18 Hz, 13^1^-CH_2_), 4.59 (1H, q, *J* = 7 Hz, 18-H), 4.40 (2H, s, NCOCH_2_), 4.37 (1H, d, *J* = 9 Hz, 17-H), 3.81 (2H, q, *J* = 8 Hz, 8-CH_2_), 3.71 (3H, s, 12-CH_3_), 3.58 (3H, s, 17^2^-COOCH_3_), 3.52 (3H, s, 2-CH_3_), 3.38 (3H, s, 7-CH_3_), 2.73–2.56, 2.33–2.24 (each 2H, m, 17-CH_2_CH_2_), 2.16 (1H, br, 3^1^-OH), 1.83 (3H, d, *J* = 7 Hz, 18-CH_3_), 1.75 (3H, t, *J* = 8 Hz, 8^1^-CH_3_), −0.74, −2.77 (each 1H, br-s, NH×2); HRMS (ESI) found: *m*/*z* = 685.3474. calcd for C_41_H_45_N_6_O_4_: [M+H]^+^, 685.3497.

**Figure S1i.** ^1^H NMR spectrum (600 MHz) of **2i** in CDCl_3_.

**Synthesis of Zn-BChl-*d* analogs 1a–i**

Zinc methyl (131*E*)-acetylhydrazinylidene-131-deoxo-3-devinyl-3-hydroxy-methyl-pyropheophorbide-*a* (**1a**): Zinc metalation of **2a** (6.1 mg) gave the titled zinc complex (0.81 mg, 1.2 μmol, 12%): dark green solid; mp = 208 °C; Vis (THF) λ_max_/nm = 649 (relative intensity 0.66), 602 (0.08), 557 ((0.04), 518 (0.05), 421 (1.00); ^1^H NMR (600 MHz, 3% C_5_D_5_N–CDCl_3_) δ/ppm = 9.64 (1H, s, 5-H), 9.58 (1H, s, 10-H), 8.92 (1H, s, 13^1^=NNH), 8.53 (1H, s, 20-H), 5.93 (2H, s, 3-CH_2_), 5.31, 5.20 (each 1H, d, *J* = 17 Hz, 13^1^-CH_2_), 4.49 (1H, br-q, *J* = 7 Hz, 18-H), 4.28 (1H, br-d, *J* = 9 Hz, 17-H), 3.83 (2H, q, *J* = 7 Hz, 8-CH_2_), 3.70 (3H, s, 12-CH_3_), 3.57 (3H, s, 17^2^-COOCH_3_), 3.41 (3H, s, 2-CH_3_), 3.32 (3H, s, 7-CH_3_), 2.65 (3H, s, 13^1^=NCOCH_3_), 2.62–2.56, 2.32–2.23, 1.97–1.91, 1.86–1.82 (each 1H, m, 17-CH_2_CH_2_), 1.74 (3H, t, *J* = 7 Hz, 8-CH_3_), 1.74 (3H, d, *J* = 7 Hz, 18-CH_3_) [The 3^1^-OH signal could be invisible.]; HRMS (ESI) found: *m*/*z* = 671.2322, calcd for C_35_H_39_N_6_O_4_Zn: [M+H]^+^, 671.2319.

**Figure S2a.** ^1^H NMR spectrum (600 MHz) of **1a** in 3% C_5_D_5_N–CDCl_3_.

Zinc methyl 131-deoxo-3-devinyl-3-hydroxymethyl-(131*E*)-propanoyl-hydrazinylidene-pyropheophorbide-*a* (**1b**): Zinc metalation of **2b** (6.2 mg) gave the titled zinc complex (1.2 mg, 1.7 μmol, 17%): dark green solid; mp = 146 °C; Vis (THF) λ_max_/nm = 648 (relative intensity 0.68), 601 (0.05), 557 (0.03), 518 (0.05), 420 (1.00); ^1^H NMR (600 MHz, 3% C_5_D_5_N–CDCl_3_) δ/ppm = 9.64 (1H, s, 5-H), 9.58 (1H, s, 10-H), 8.87 (1H, s, 13^1^=NNH), 8.53 (1H, s, 20-H), 5.93 (2H, s, 3-CH_2_), 5.31, 5.20 (each 1H, d, *J* = 18 Hz, 13^1^-CH_2_), 4.49 (1H, dq, *J* = 2, 7 Hz, 18-H), 4.28 (1H, dt, *J* = 8, 2 Hz, 17-H), 3.84 (2H, q, *J* = 7.5 Hz, 8-CH_2_), 3.70 (3H, s, 12-CH_3_), 3.57 (3H, s, 17^2^-COOCH_3_), 3.42 (3H, s, 2-CH_3_), 3.33 (3H, s, 7-CH_3_), 3.08 (2H, q, *J* = 7.5 Hz, NCOCH_2_), 2.61–2.57, 2.45–2.40, 1.97–1.92, 1.89–1.84 (each 1H, m, 17-CH_2_CH_2_), 1.74 (3H, t, *J* = 7.5 Hz, 8^1^-CH_3_), 1.74 (3H, d, *J* = 7 Hz, 18-CH_3_), 1.42 (3H, t, *J* = 7.5 Hz, NCOCCH_3_) [The 3^1^-OH signal could be invisible.]; HRMS (ESI) found: *m*/*z* = 684.2374, calcd for C_36_H_40_N_6_O_4_Zn: M^+^, 684.2379.

**Figure S2b.** ^1^H NMR spectrum (600 MHz) of **1b** in 3% C_5_D_5_N–CDCl_3_.

Zinc methyl (131*E*)-butanoylhydrazinylidene-131-deoxo-3-devinyl-3-hydroxy-methyl-pyropheophorbide-*a* (**1c**): Zinc metalation of **2c** (6.4 mg) gave the titled zinc complex (1.1 mg, 1.6 μmol, 16%): dark green solid; mp = 160 °C; Vis (THF) λ_max_/nm = 647 (relative intensity 0.71), 601 (0.07), 555 (0.03), 519 (0.05), 420 (1.00); ^1^H NMR (600 MHz, 3% C_5_D_5_N–CDCl_3_) δ/ppm = 9.64 (1H, s, 5-H), 9.58 (1H, s, 10-H), 9.00 (1H, s, 13^1^=NNH), 8.53 (1H, s, 20-H), 5.93 (2H, s, 3-CH_2_), 5.31, 5.20 (each 1H, d, *J* = 17.5 Hz, 13^1^-CH_2_), 4.49 (1H, dq, *J* = 2, 7 Hz, 18-H), 4.27 (1H, dt, *J* = 8, 2 Hz, 17-H), 3.83 (2H, q, *J* = 7.5 Hz, 8-CH_2_), 3.70 (3H, s, 12-CH_3_), 3.58 (3H, s, 17^2^-COOCH_3_), 3.42 (3H, s, 2-CH_3_), 3.33 (3H, s, 7-CH_3_), 3.05, 3.02 (each 1H, dt, *J* = 14.5, 7.5 Hz, NCOCH_2_), 2.63–2.57, 2.47–2.42, 2.27–2.21, 1.98–1.93 (each 1H, m, 17-CH_2_CH_2_), 1.97 (2H, sextet, *J* = 7.5 Hz, NCOCCH_2_), 1.74 (3H, t, *J* = 7.5 Hz, 8^1^-CH_3_), 1.73 (3H, d, *J* = 7 Hz, 18-CH_3_), 1.19 (3H, t, *J* = 7.5 Hz, NCOC_2_CH_3_) [The 3^1^-OH signal could be invisible.]; HRMS (ESI) found: *m*/*z* = 698.2554 and 699.2633, calcd for C_37_H_42_N_6_O_4_Zn: M^+^, 698.2554 and C_37_H_43_N_6_O_4_Zn: [M+H]^+^, 699.2632.

**Figure S2c.** ^1^H NMR spectrum (600 MHz) of **1c** in 3% C_5_D_5_N–CDCl_3_.

Zinc methyl 131-deoxo-3-devinyl-3-hydroxymethyl-(131*E*)-pentanoyl-hydrazinylidene-pyropheophorbide-*a* (**1d**): Zinc metalation of **2d** (6.5 mg) gave the titled zinc complex (2.6 mg, 3.6 μmol, 36%): dark green solid; mp = 162 °C; Vis (THF) λ_max_/nm = 647 (relative intensity 0.71), 601 (0.06), 555 (0.02), 518 (0.04), 420 (1.00); ^1^H NMR (600 MHz, 3% C_5_D_5_N–CDCl_3_) δ/ppm = 9.64 (1H, s, 5-H), 9.58 (1H, s, 10-H), 8.88 (1H, s, 13^1^=NNH), 8.53 (1H, s, 20-H), 5.92 (2H, s, 3-CH_2_), 5.31, 5.20 (each 1H, d, *J* = 18 Hz, 13^1^-CH_2_), 4.49 (1H, dq, *J* = 2, 7 Hz, 18-H), 4.28 (1H, dt, *J* = 8, 2 Hz, 17-H), 3.84 (2H, q, *J* = 7.5 Hz, 8-CH_2_), 3.70 (3H, s, 12-CH_3_), 3.57 (3H, s, 17^2^-COOCH_3_), 3.42 (3H, s, 2-CH_3_), 3.33 (3H, s, 7-CH_3_), 3.07, 3.04 (each 1H, dt, *J* = 15, 7.5 Hz, NCOCH_2_), 2.62–2.56, ≈2.3, 1.97–1.90, 1.87–1.84 (each 1H, m, 17-CH_2_CH_2_), 1.93 (2H, quintet, *J* = 7.5 Hz, NCOCCH_2_), 1.74 (3H, t, *J* = 7.5 Hz, 8^1^-CH_3_), 1.74 (3H, t, *J* = 7 Hz, 18-CH_3_), 1.61 (2H, sextet, *J* = 7 Hz, NCOC_2_CH_2_), 1.09 (3H, t, *J* = 7 Hz, NCOC_3_CH_3_) [The 3^1^-OH signal could be invisible.]; HRMS (ESI) found: *m*/*z* = 713.2790, calcd for C_38_H_45_N_6_O_4_Zn: [M+H]^+^, 713.2788.

**Figure S2d.** ^1^H NMR spectrum (600 MHz) of **1d** in 3% C_5_D_5_N–CDCl_3_.

Zinc methyl 131-deoxo-3-devinyl-(131*E*)-hexanoylhydrazinylidene-3-hydroxy-methyl-pyropheophorbide-*a* (**1e**): Zinc metalation of **2e** (6.6 mg) gave the titled zinc complex (1.0 mg, 1.4 μmol, 14%): dark green solid; mp = 158 °C; Vis (THF) λ_max_/nm = 647 (relative intensity 0.71), 601 (0.06), 554 (0.02), 517 (0.04), 420 (1.00); ^1^H NMR (600 MHz, 3% C_5_D_5_N–CDCl_3_) δ/ppm = 9.64 (1H, s, 5-H), 9.58 (1H, s, 10-H), 8.88 (1H, s, 13^1^=NNH), 8.53 (1H, s, 20-H), 5.93 (2H, s, 3-CH_2_), 5.31, 5.20 (each 1H, d, *J* = 18 Hz, 13^1^-CH_2_), 4.49 (1H, dq, *J* = 2, 7 Hz, 18-H), 4.28 (1H, dt, *J* = 8, 2 Hz, 17-H), 3.84 (2H, q, *J* = 7 Hz, 8-CH_2_), 3.70 (3H, s, 12-CH_3_), 3.57 (3H, s, 17^2^-COOCH_3_), 3.42 (3H, s, 2-CH_3_), 3.33 (3H, s, 7-CH_3_), 3.06, 3.03 (each 1H, dt, *J* = 15, 7.5 Hz, NCOCH_2_), 2.63–2.57, 2.46–2.41, 1.98–1.94, 1.86–1.84 (each 1H, m, 17-CH_2_CH_2_), 1.95 (2H, quintet, *J* = 7 Hz, NCOCCH_2_), 1.74 (3H, t, *J* = 7 Hz, 8^1^-CH_3_), 1.74 (3H, d, *J* = 7 Hz, 18-CH_3_), 1.59–1.49 (4H, m, NCOC_2_CH_2_CH_2_), 1.01 (3H, t, *J* = 7 Hz, NCOC_4_CH_3_) [The 3^1^-OH signal could be invisible.]; HRMS (ESI) found: *m*/*z* = 726.2887 and 727.2953, calcd for C_39_H_46_N_6_O_4_Zn: M^+^, 726.2867 and C_39_H_47_N_6_O_4_Zn: [M+H]^+^, 727.2945.

**Figure S2e.** ^1^H NMR spectrum (600 MHz) of **1e** in 3% C_5_D_5_N–CDCl_3_.

Zinc methyl 131-deoxo-3-devinyl-(131*E*)-heptanoylhydrazinylidene-3-hydroxy-methyl-pyropheophorbide-*a* (**1f**): Zinc metalation of **2f** (6.8 mg) gave the titled zinc complex (1.3 mg, 1.8 μmol, 18%): dark green solid; mp = 162 °C; Vis (THF) λ_max_/nm = 647 (relative intensity 0.70), 600 (0.06), 555 (0.02), 517 (0.04), 419 (1.00); ^1^H NMR (600 MHz, 3% C_5_D_5_N–CDCl_3_) δ/ppm = 9.64 (1H, s, 5-H), 9.58 (1H, s, 10-H), 8.92 (1H, s, 13^1^=NNH), 8.53 (1H, s, 20-H), 5.93 (2H, s, 3-CH_2_), 5.30, 5.20 (each 1H, d, *J* = 17.5 Hz, 13^1^-CH_2_), 4.49 (1H, dq, *J* = 2, 7 Hz, 18-H), 4.27 (1H, dt, *J* = 9, 2 Hz, 17-H), 3.84 (2H, q, *J* = 7.5 Hz, 8-CH_2_), 3.70 (3H, s, 12-CH_3_), 3.57 (3H, s, 17^2^-COOCH_3_), 3.42 (3H, s, 2-CH_3_), 3.33 (3H, s, 7-CH_3_), 3.06, 3.03 (each 1H, dt, *J* = 14.5, 7.5 Hz, NCOCH_2_), 2.62–2.53, 2.45–2.40, 2.29–2.23, 1.96–1.88 (each 1H, m, 17-CH_2_CH_2_), 1.94 (2H, quintet, *J* = 7.5 Hz, NCOCCH_2_), 1.74 (3H, t, *J* = 7.5 Hz, 8^1^-CH_3_), 1.73 (3H, d, *J* = 7 Hz, 18-CH_3_), 1.59 (2H, quintet, *J* = 7 Hz, NCOC_2_CH_2_), 1.47 (2H, quintet, *J* = 7 Hz, NCOC_3_CH_2_), 1.44 (2H, sextet, *J* = 7 Hz, NCOC_4_CH_2_), 0.97 (3H, t, *J* = 7 Hz, NOC_5_CH_3_) [The 3^1^-OH signal could be invisible.]; HRMS (ESI) found: *m*/*z* = 740.3028 and 741.3099, calcd for C_40_H_48_N_6_O_4_Zn: M^+^, 740.3023 and C_40_H_49_N_6_O_4_Zn: [M+H]^+^, 741.3101.

**Figure S2f.** ^1^H NMR spectrum (600 MHz) of **1f** in 3% C_5_D_5_N–CDCl_3_.

Zinc methyl 131-deoxo-3-devinyl-3-hydroxymethyl-(131*E*)-(2-methylpropanoyl)-hydrazinylidene-pyropheophorbide-*a* (**1g**): Zinc metalation of **2g** (6.4 mg) gave the titled zinc complex (3.5 mg, 5.0 μmol, 50%): dark green solid; mp = 195 °C; Vis (THF) λ_max_/nm = 648 (relative intensity 0.70), 601 (0.07), 555 (0.03), 518 (0.04), 420 (1.00); ^1^H NMR (600 MHz, 3% C_5_D_5_N–CDCl_3_) δ/ppm = 9.64 (1H, s, 5-H), 9.59 (1H, s, 10-H), 8.83 (1H, s, 13^1^=NNH), 8.53 (1H, s, 20-H), 5.93 (2H, s, 3-CH_2_), 5.32, 5.21 (each 1H, d, *J* = 17 Hz, 13^1^-CH_2_), 4.50 (1H, dq, *J* = 2, 7 Hz, 18-H), 4.28 (1H, dt, *J* = 8, 2 Hz, 17-H), 3.85 (1H, septet, *J* = 7 Hz, NCOCH), 3.84 (2H, q, *J* = 7.5 Hz, 8-CH_2_), 3.70 (3H, s, 12-CH_3_), 3.56 (3H, s, 17^2^-COOCH_3_), 3.41 (3H, s, 2-CH_3_), 3.32 (3H, s, 7-CH_3_), 2.62–2.57, 2.44–2.39, 2.32–2.25, 1.95–1.90 (each 1H, m, 17-CH_2_CH_2_), 1.74 (3H, t, *J* = 7.5 Hz, 8^1^-CH_3_), 1.74 (3H, d, *J* = 7 Hz, 18-CH_3,_), 1.45, 1.44 (each 3H, t, *J* = 7 Hz, NCOC(CH_3_)_2_) [The 3^1^-OH signal could be invisible.]; HRMS (ESI) found: *m*/*z* = 699.2633, calcd for C_37_H_43_N_6_O_4_Zn: [M+H]^+^, 699.2632.

**Figure S2g.** ^1^H NMR spectrum (600 MHz) of **1g** in 3% C_5_D_5_N–CDCl_3_.

Zinc methyl 131-deoxo-3-devinyl-(131*E*)-(2,2-dimethylpropanoyl)-hydrazinylidene-3-hydroxymethyl-pyropheophorbide-*a* (**1h**): Zinc metalation of **2h** (6.5 mg) gave the titled zinc complex (2.1 mg, 3.0 μmol, 30%): dark green solid; mp = 162 °C; Vis (THF) λ_max_/nm = 648 (relative intensity 0.69), 601 (0.07), 557 (0.02), 519 (0.04), 420 (1.00); ^1^H NMR (600 MHz, 3% C_5_D_5_N–CDCl_3_) δ/ppm = 9.65 (1H, s, 5-H), 9.56 (1H, s, 10-H), 9.06 (1H, s, 13^1^=NNH), 8.52 (1H, s, 20-H), 5.92 (2H, s, 3-CH_2_), 5.45, 5.30 (each 1H, d, *J* = 17.5 Hz, 13^1^-CH_2_), 4.47 (1H, br-q, *J* = 7 Hz, 18-H), 4.27 (1H, br-d, *J* = 8 Hz, 17-H), 3.82 (2H, q, *J* = 8 Hz, 8-CH_2_), 3.77 (3H, s, 12-CH_3_), 3.59 (3H, s, 17^2^-COOCH_3_), 3.41 (3H, s, 2-CH_3_), 3.31 (3H, s, 7-CH_3_), 2.65–2.58, 2.51–2.44, 2.24–2.18, 1.96–1.90 (each 1H, m, 17-CH_2_CH_2_), 1.71 (3H, t, *J* = 8 Hz, 8^1^-CH_3_), 1.71 (3H, d, *J* = 7 Hz, 18-CH_3_), 1.52 (9H, s, C(CH_3_)_3_) [The 3^1^-OH signal could be invisible.]; HRMS (ESI) found: *m*/*z* = 712.2686 and 713.2776, calcd for C_38_H_44_N_6_O_4_Zn: M^+^, 712.2710 and C_38_H_45_N_6_O_4_Zn: [M+H]^+^, 713.2788.

**Figure S2h.** ^1^H NMR spectrum (600 MHz) of **1h** in 3% C_5_D_5_N–CDCl_3_.

Zinc methyl 131-deoxo-3-devinyl-3-hydroxymethyl-(131*E*)-(phenylacetyl)-hydrazinylidene-pyropheophorbide-*a* (**1i**): Zinc metalation of **2i** (6.8 mg) gave the titled zinc complex (3.2 mg, 4.3 μmol, 43%): dark green solid; mp = 164° C; Vis (THF) λ_max_/nm = 648 (relative intensity 0.71), 602 (0.07), 554 (0.03), 518 (0.05), 420 (1.00); ^1^H NMR (600 MHz, 3% C_5_D_5_N–CDCl_3_) δ/ppm = 9.65 (1H, s, 5-H), 9.58 (1H, s, 10-H), 9.01 (1H, s, 13^1^=NNH), 8.52 (1H, s, 20-H), 7.65 (2H, d, *J* = 7.5 Hz, *o*-H of Ph), 7.41 (2H, t, *J* = 7.5 Hz, *m*-H of Ph), 7.30 (1H, t, *J* = 7.5 Hz, *p*-H of Ph), 5.92 (2H, s, 3-CH_2_), 5.31, 5.20 (each 1H, d, *J* = 18 Hz, 13^1^-CH_2_), 4.48 (1H, dq, *J* = 2, 7 Hz, 18-H), 4.42, 4.38 (1H, d, *J* = 14 Hz, 13^1^-NNCOCH_2_), 4.26 (1H, dt, *J* = 9, 2 Hz, 17-H), 3.84 (2H, q, *J* = 8 Hz, 8-CH_2_), 3.75 (3H, s, 12-CH_3_), 3.56 (3H, s, 17^2^-COOCH_3_), 3.41 (3H, s, 2-CH_3_), 3.33 (3H, s, 7-CH_3_), 2.61–2.55, 2.44–2.39, ≈2.3, 1.97–1.91 (each 1H, m, 17-CH_2_CH_2_), 1.74 (3H, t, *J* = 8 Hz, 8^1^-CH_3_), 1.73 (3H, d, *J* = 7 Hz, 18-CH_3_) [The 3^1^-OH signal could be invisible.]; HRMS (ESI) found: *m*/*z* = 746.2549 and 747.2634, calcd for C_41_H_42_N_6_O_4_Zn: M^+^, 746.2554 and C_41_H_43_N_6_O_4_Zn: [M+H]^+^,747.2632.

**Figure S2i.** ^1^H NMR spectrum (600 MHz) of **1i** in 3% C_5_D_5_N–CDCl_3_.

**Table S1.** Total energy (kcal/mol) of (13^1^*E*/*Z*)-**1** (zinc complex) and **2** (free base) estimated by MM+/PM3 calculation.^a^

Compound (13^1^*E*)-form (13^1^*Z*)-form Difference^b^

**1a** (acetyl) 151.16 154.35 −3.19

**1b** (propanoyl) 152.43 155.67 −3.24

**1c** (butanoyl) 153.03 156.30 −3.27

**1d** (pentanoyl) 153.66 156.93 −3.27

**1e** (hexanoyl) 154.28 157.56 −3.28

**1f** (heptanoyl) 154.92 158.20 −3.28

**1g** (isobutyryl) 154.20 157.48 −3.28

**1h** (pivaloyl) 156.94 160.40 −3.46

**1i** (phenylacetyl) 149.03 152.27 −3.24

**2a** (acetyl) 62.33 65.85 −3.52

**2b** (propanoyl) 63.66 67.20 −3.54

**2c** (butanoyl) 64.26 67.83 −3.57

**2d** (pentanoyl) 64.88 68.47 −3.59

**2e** (hexanoyl) 65.51 69.11 −3.60

**2f** (heptanoyl) 66.14 69.74 −3.60

**2g** (isobutyryl) 65.41 69.03 −3.62

**2h** (pivaloyl) 68.26 71.93 −3.67

**2i** (phenylacetyl) 60.24 63.79 −3.55

^a^ The calculation was based on the procedures reported in Kureishi, Y. and H. Tamiaki (1982) Synthesis and self-aggregation of zinc 20-halogenochlorins as a model for bacteriochlorophylls *c*/*d*. *J. Porphyrins Phthalocyanines* **2**, 159–169.

^b^ Total energy of (13^1^*E*)-form − total energy of (13^1^*Z*)-form.

**Table S2.** Total energy (kcal/mol) of *cis*- and *trans*-conformers around the amide bond in (13^1^*E*)-**1** (zinc complex) and **2** (free base) estimated by MM+/PM3 calculation.^a^

Compound *Cis*-conformer *Trans*-conformer Difference^b^

**1a** (acetyl) 151.16 153.14 −1.98

**1b** (propanoyl) 152.43 154.62 −2.19

**1c** (butanoyl) 153.03 155.19 −2.16

**1d** (pentanoyl) 153.66 155.92 −2.26

**1e** (hexanoyl) 154.28 156.51 −2.23

**1f** (heptanoyl) 154.92 157.12 −2.20

**1g** (isobutyryl) 154.20 155.99 −1.79

**1h** (pivaloyl) 156.94 157.63 −0.69

**1i** (phenylacetyl) 149.03 151.40 −2.37

**2a** (acetyl) 62.33 64.51 −2.18

**2b** (propanoyl) 63.66 65.87 −2.21

**2c** (butanoyl) 64.26 66.56 −2.30

**2d** (pentanoyl) 64.88 67.16 −2.28

**2e** (hexanoyl) 65.51 67.87 −2.36

**2f** (heptanoyl) 66.14 68.49 −2.35

**2g** (isobutyryl) 65.41 67.36 −1.95

**2h** (pivaloyl) 68.26 69.00 −0.74

**2i** (phenylacetyl) 60.24 62.70 −2.46

^a^ The calculation was based on the procedures reported in Kureishi, Y. and H. Tamiaki (1982) Synthesis and self-aggregation of zinc 20-halogenochlorins as a model for bacteriochlorophylls *c*/*d*. *J. Porphyrins Phthalocyanines* **2**, 159–169.

^b^ Total energy of *cis*-conformer − total energy of *trans*-conformer.


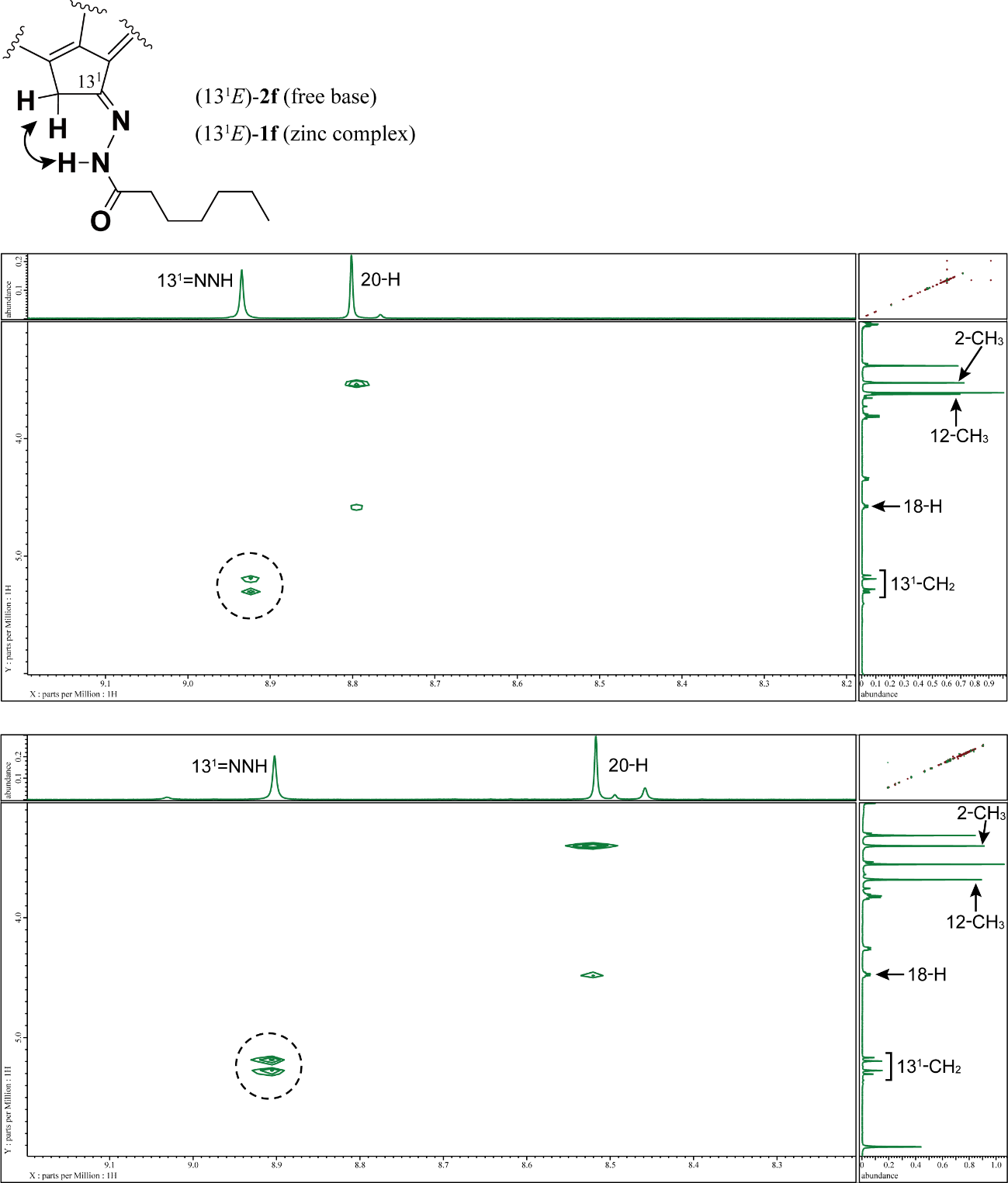


**Figure S3.** NOESY of **2f** in CDCl_3_ (upper) and **1f** in 3% C_5_D_5_N–CDCl_3_ (lower) at a specific region.
